# Comparative immunohistochemical expression of Beta catenin and CD163 between dysplastic / non-dysplastic oral lichen planus and lichenoid lesions (EX-VIVO STUDY)

**DOI:** 10.1186/s12903-024-04822-5

**Published:** 2024-09-26

**Authors:** Heba Ahmed Saleh, Ghada Nabil, Sarah Ahmed Mohammed Mahmoud Badawy

**Affiliations:** 1 Oral & Maxillofacial Pathology, Faculty of Dentistry, Cairo University, Cairo, Egypt; 2 Oral Medicine, Faculty of Dentistry, Cairo University, Cairo, Egypt; 3 11 el Saraya Street, Manyal, Cairo, Egypt

**Keywords:** Β-catenin, CD163, M2 macrophages, TAM, TAM-specific β-catenin signaling, Dysplastic, Non-dysplastic OLP, Dysplastic, Non-dysplastic OLL, OMPD

## Abstract

**Background:**

Oral lichen planus is a well-known chronic inflammatory mucocutaneous disorder, which has clinical and histological presentation that mimics oral lichenoid reaction. According to the fifth edition of WHO, both conditions are considered as oral potentially malignant disorders. Recent studies on oral potential disorders documented deregulation of some signaling molecules related to the Wnt/β-catenin pathway. Therefore this study aimed to compare the immune expression of β-catenin & CD163 in dysplastic /non-dysplastic cases of Oral lichen planus & oral lichenoid lesion. In addition, a statistical correlation between both immune markers was done regardless of the type of the study group.

**Methods:**

Four study groups were designated as 2 groups of Oral lichen planus (one dysplastic & one non –dysplastic) and the other 2 groups were oral lichenoid lesions (one dysplastic & one non –dysplastic). Ten cases in each group were collected and investigated by immunohistochemistry. The area percent of beta catenin and also counting of m2 macrophages expressing + CD163 marker was calculated in the study groups.

**Results:**

The Statistical analysis highlighted a statistically significant difference between the studied groups. Moreover, Pearson correlation test reported a significant moderate positive correlation between beta catenin & CD163 expression in the studied cases.

**Conclusion:**

Our findings supported new perceptions of the mechanism by which tumor associated macrophage specific β-catenin signaling promotes the aggressive behavior of oral potential malignant disorders.

**Clinical relevance:**

Evidence of the relationship between beta catenin and M2 macrophages (+ CD163) may enhance the development of macrophage-based strategies for treatment and improve the prognosis of such cases.

## Introduction

Oral lichen planus (OLP) is a well-known chronic inflammatory mucocutaneous disorder with estimated global prevalence about 0.5–2.6%. Its clinically erosive form usually manifests as a bilateral white keratotic lines with reticular pattern (Wickham striae) in an erosive bed [1]. On the other hand, oral lichenoid lesions (OLL) may clinically present as a unilateral lesion, rather than bilateral lesions, and may be associated with the use of several dental materials and medications, such as antihypertensives, nonsteroidal anti- inflammatory drugs, anticonvulsants, and antimalarials. Although the microscopic features of OLL overlap with those of OLP to a great, some differences are histologically reported in OLL as higher number of apoptotic keratinocytes, more diffused and deeper sub-epithelial inflammation composed of mixture of plasma and eosinophil cells [2].

According to the fifth edition of World Health Organization (WHO), both OLP&OLL are considered as oral potentially malignant disorders (OPMDs) [3]. Epithelial dysplasia may be histologically detected in these cases and conversion of those lesions into oral squamous cell carcinoma (OSCC) may occur [1]. According to the current literature, the mechanism of their malignant transformation is still unidentified. It has been suggested that the release of inflammatory mediators during their inflammatory process, allow genetic changes in the epithelial cells. This leads to epithelial dysplastic changes, which in conclusion accelerates their malignant transformation [2].

Recent studies on OPMDs documented deregulation of some signaling molecules related to the Wnt/β-catenin pathway. This signaling pathway is previously documented to be involved in several biological functions, including cell regeneration, differentiation and proliferation [4]. Macrophages have important immune functions in the living body such as recognizing and killing pathogens, initiating and resolving inflammation, and controlling the adaptive immune system. Macrophages undergo polarization into two main groups: classically activated macrophages (M1), which lead to pro-inflammatory responses, and secondly, alternatively activated macrophages (M2) that govern tissue remodeling. M2 macrophages are known to be involved in the aggressiveness of various cancers, and it was revealed that CD163 + in tumor associated macrophages (TAM) predict poor prognosis in cancer patients [5]. Polarization process of macrophages is usually controlled by transcriptional changes after the exposure of different stimuli [6]. Therefore, this study is aiming to assess and compare the immunohistochemical expression of β-catenin and /M2 macrophages expressing CD163 in dysplastic versus non-dysplastic cases of OLP and OLL.

## Methodology

All experimental protocols were approved by the research ethics committee, Faculty of Dentistry, Cairo University, registered by no. (19 12 22), to use the archival blocks of cases; that were diagnosed OLP and OLL in the last 10 years retrieved from Oral and Maxillofacial Pathology Department, Faculty of Dentistry, Cairo University. No informed consent from participants was needed, as the current study was performed on archival wax blocks. The study was conducted in compliance with the Guidelines documented in Remark checklist of using biological markers on excised tissues [7]. Cases that received corticosteroid treatment, and those had incomplete personal/medical status were excluded from the study. 40 cases were included in this study and divided into 4 groups (dysplastic OLP, dysplastic OLL, non-dysplastic OLP & non-dysplastic OLL); 10 cases per each group. All cases were stained with H&E and rediagnosed (after long period from the initial diagnosis) based on the latest WHO histological criteria by two blinded pathologists. The following criteria were considered during rediagnosis of the study cases; to resolve any inter-observer variability: site of the lesion (either unilateral or bilateral), the documented history of contact allergy or hypersensitivity after drug administration, type of inflammatory subepithelial infiltration (either composed of lymphocytes only or mixed types of inflammatory cells) and finally the detection of deep perivascular inflammatory infiltrate [8]. Stratification of cases into dysplastic and non-dysplastic was histologically done according to the presence of signs of epithelial dysplasia as described in the latest WHO classification of tumors of oral cavity [3].

β-catenin (E-5): sc-7963 and CD163 (ED2): sc-58,965 primary antibodies (Santa Cruz Biotechnology Inc., Dallas, TX, USA) [9]. Three-micrometer sections were cut from the paraffin-embedded tissue blocks for immunohistochemistry protocol according to Lin and Chen, (2014) [10]. Both immune markers were utilized fully automated on a Ventana Benchmark Ultra platform following the Ultraview DAB detection kit procedure (cat #760 − 500, Ventana Medical Systems/Roche Diagnostics).

Immunostained sections were examined using high power fields (X200) by light microscope; SOPTOP EX20 biological microscope (Sunny Optical technology co., Zhejiang, China) and HD digital camera model no. XCAM1080PHB (ToupTek, China), and the most homogenous areas of the positive reaction were chosen for evaluation. The image analyzer computer system applying Imagej 1.53e software (USA) was used for automated measurement of area percent of β-catenin positivity in epithelium. QuPath-0.3.2 image analyzer software (UK), was used for manual counting of CD163 positive cells in stroma, cytoplasmic and nuclear positive cells for β-catenin in epithelium. It was performed in a standard frame area of 5.04 × 106 µm^2^, five fields were measured per case. Capturing microscopic images and all mentioned measurements were performed blindly by an expert in Analytica Research Center, El Haram, Cairo, Egypt, using SOPTOP EX20 biological microscope (Sunny Optical technology co., Zhejiang, China) and HD digital camera model no. XCAM1080PHB (ToupTek, China) and the Image view software used at x40, x100 and x200 magnification powers.

### Statistical analysis

Our research hypothesis is to investigate if there is a statistical significance difference between the 4 study groups regarding the CD163 and also the β-catenin immunohistochemical expressions. The Shapiro-Wilk test of normality was used to test the normality hypothesis of variables. One-way analysis of variance (ANOVA) test was used for comparison between the groups followed by Tukey’s post hoc for multiple 2-groups comparisons. Significance level P values less than 0.05 was considered statistically significant. Pearson correlation coefficient calculator was used to evaluate the correlation between β-catenin and CD163 expressions. All statistical calculations tests were done using computer program IBM SPSS (Statistical Package for the Social Science; IBM Corp, Armonk, NY, USA) release 25 for Microsoft Windows.

## Results

**Fig. 1:**
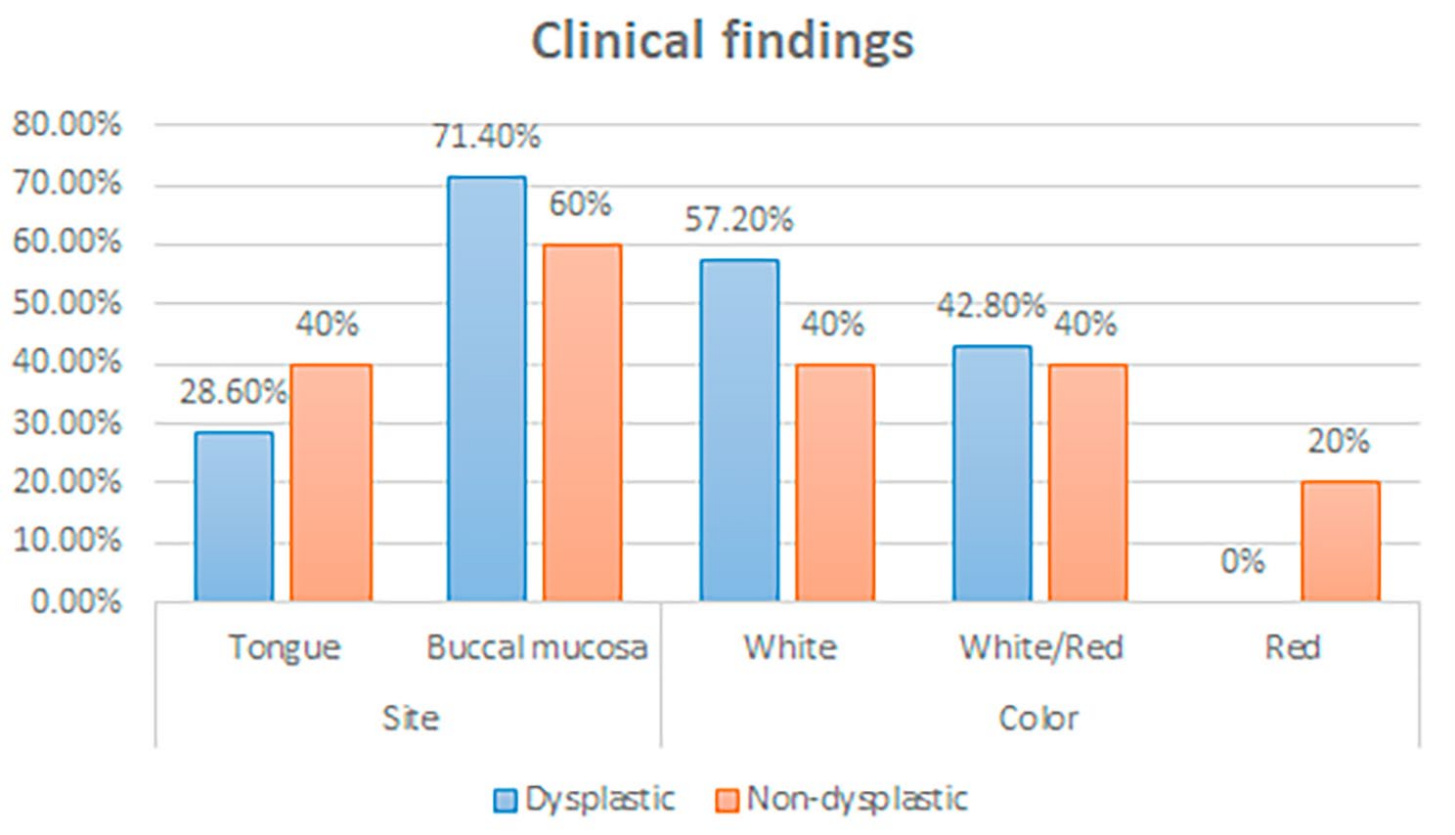
Bar chart showing the clinical findings of the studied cases

Analysis of the clinical features of the studied cases showed that two thirds of the cases occurred on the buccal mucosa, with the remaining third occurring on the tongue. The age of the patients was mostly above 45 y; with mean of 56 y ± 9.05 (46-67y). There was a strong male predilection with 90% of the cases being male. All cases underwent a follow up period from 3 to 5 years according to their reported clinical findings according to the recent treatment trends for cases of OLP and OLL [11]. The distribution and color of the lesions were clarified in (Fig. 1).

**Fig. 2:**
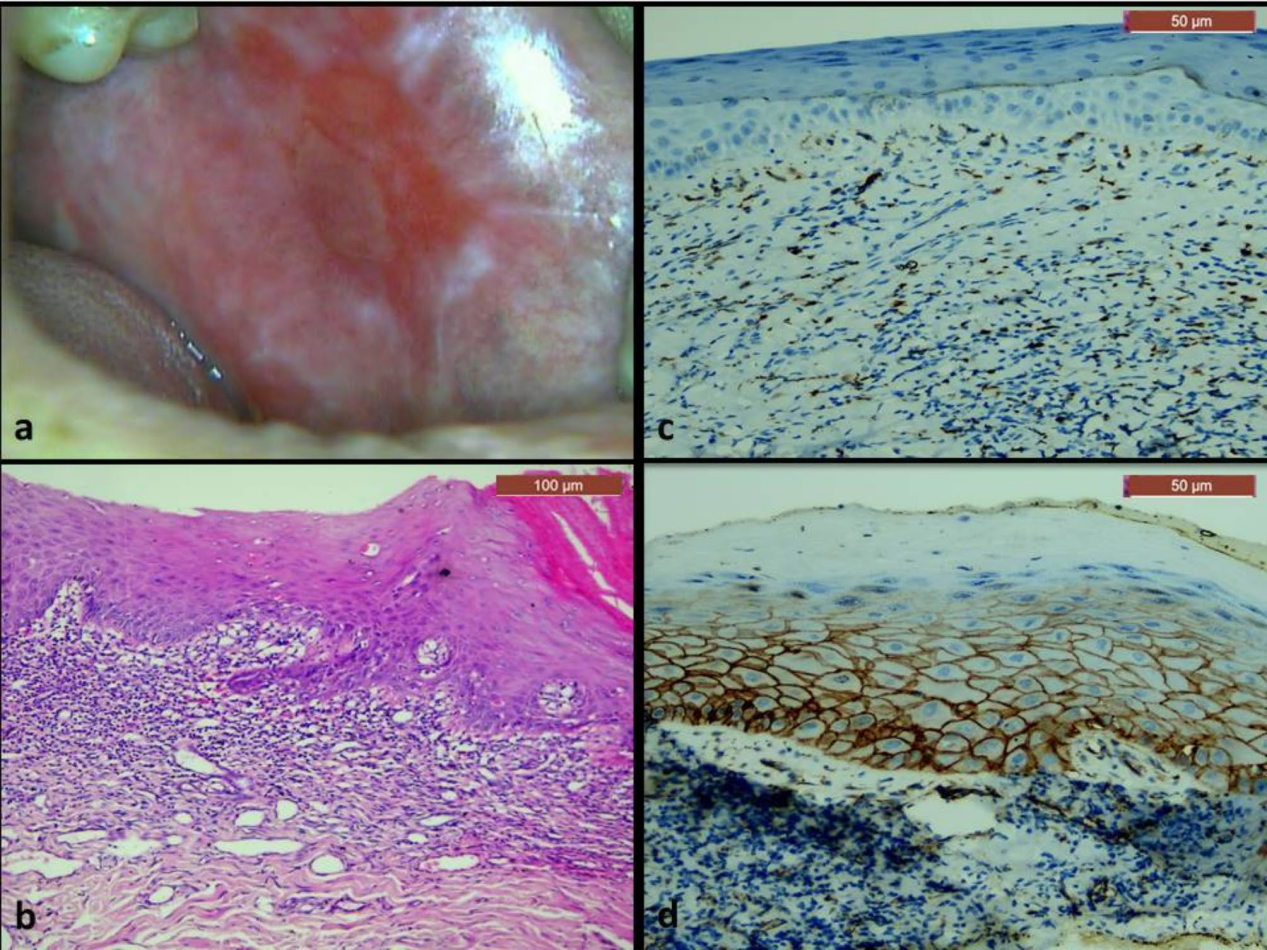
A case of non-dysplastic OLP: (**a**) clinical image showing atrophic erythematous areas on the buccal mucosa, which were surrounded by white striae. (**b**) Microscopic image showing hyperplastic stratified squamous epithelium with a prominent sub-epithelial lymphocytic band. Basal cell degeneration and civatte bodies (arrows) were evident (H&E, x100). (**c**) Immunohistochemical expression of CD163 showing cytoplasmic and nuclear expression in scattered cells in the connective tissue (x200). (**d**) Immunohistochemical expression of β-catenin showing strong membranous expression in epithelial cells with some cytoplasmic expression that was confined in the basal and parabasal cell layers (x200).

**Fig. 3:**
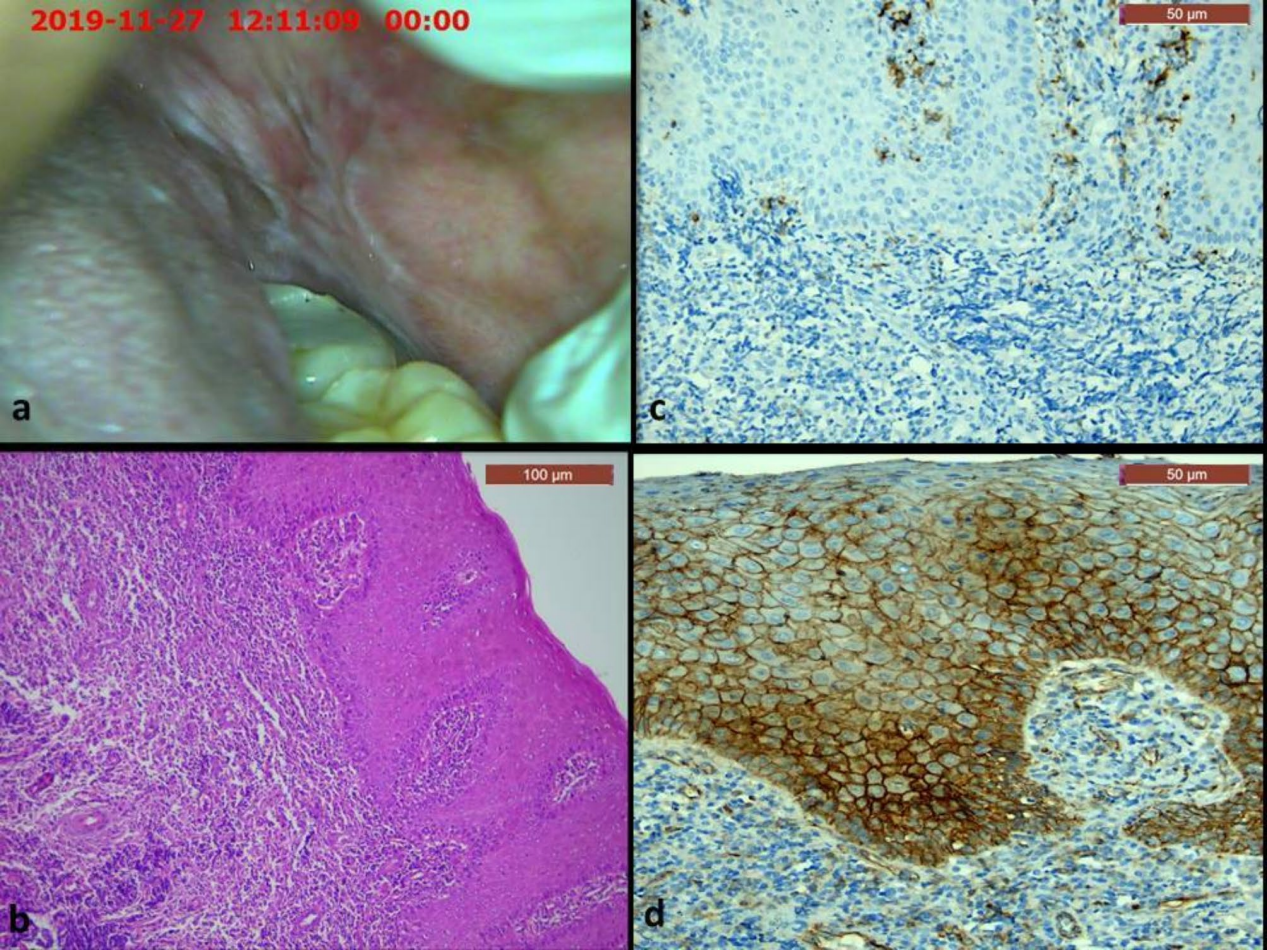
A case of non-dysplastic OLL (**a**) clinical image showing white lesion on the buccal mucosa in relation to a restoration and tiny areas of ulceration were evident intervening with the reticular pattern of the lesion. (**b**) Microscopic image showing hyperplastic stratified squamous epithelium with intense inflammation of the underlying stroma that extended deeply in the submucosa and around blood vessels (H&E, x100) (**c**) Immunohistochemical expression of CD163 showing cytoplasmic and nuclear expression in scattered cells in the connective tissue just beneath the epithelium (x200) (**d**) Immunohistochemical expression of β-catenin showing strong membranous expression in epithelial cells with some cytoplasmic expression in the basal and suprabasal cells (x200)

**Fig. 4:**
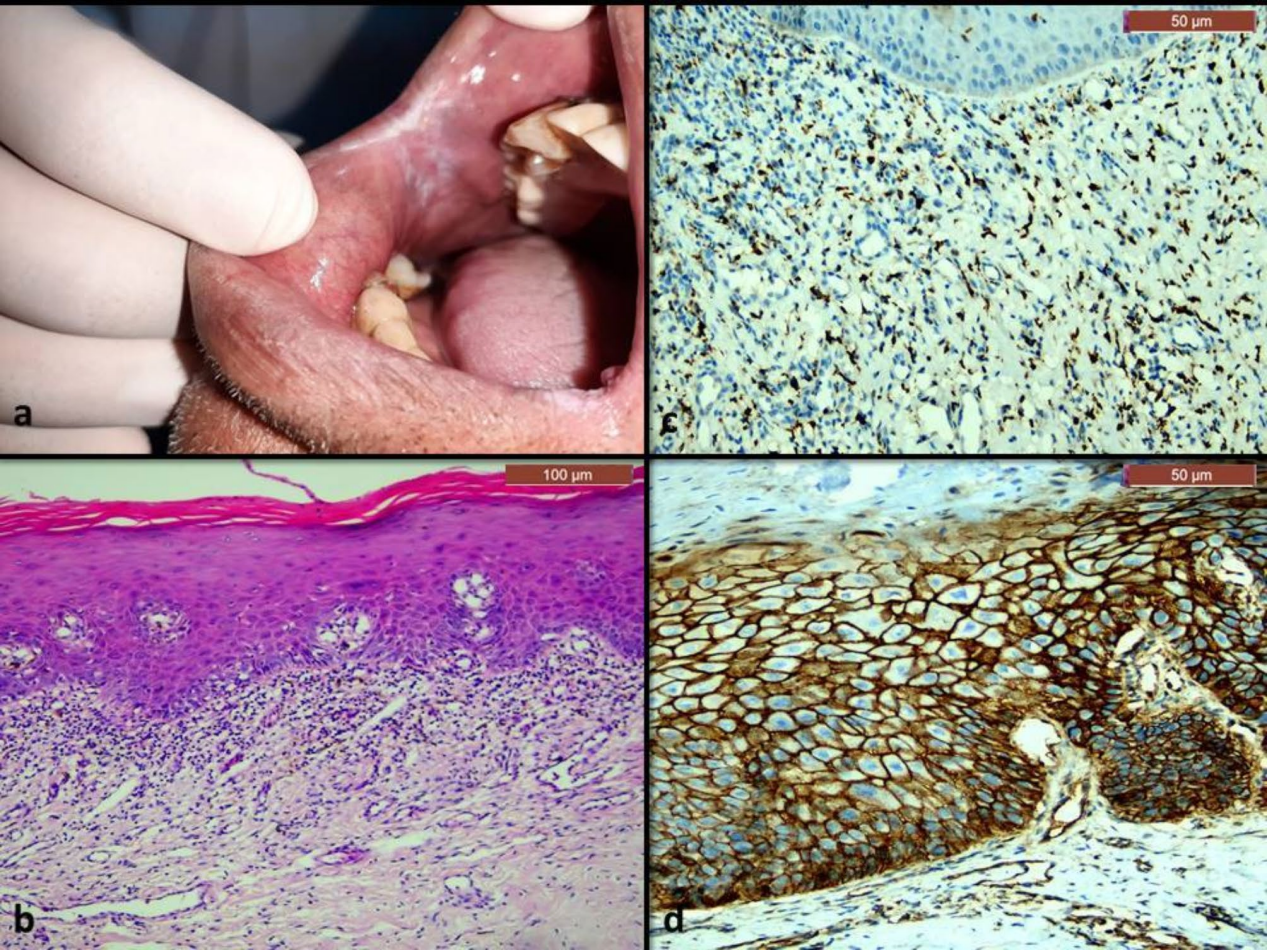
A case of dysplastic OLP: (**a**) clinical image showing white keratotic lesion on the commissure of the mouth with central ulceration (**b**) Microscopic image showing hyperplastic stratified squamous epithelium which showed cellular and nuclear pleomorphism (arrows) as well as basal cell degeneration (H&E, x100) (**c**) Immunohistochemical expression of CD163 showing cytoplasmic and nuclear expression in numerous cells in the connective tissue (x200) (**d**) Immunohistochemical expression of β-catenin showing strong membranous expression in epithelial cells with cytoplasmic expression in scattered cells in all layers of epithelium, and few nuclear expressions in the basal cell layer (x200)

**Fig. 5:**
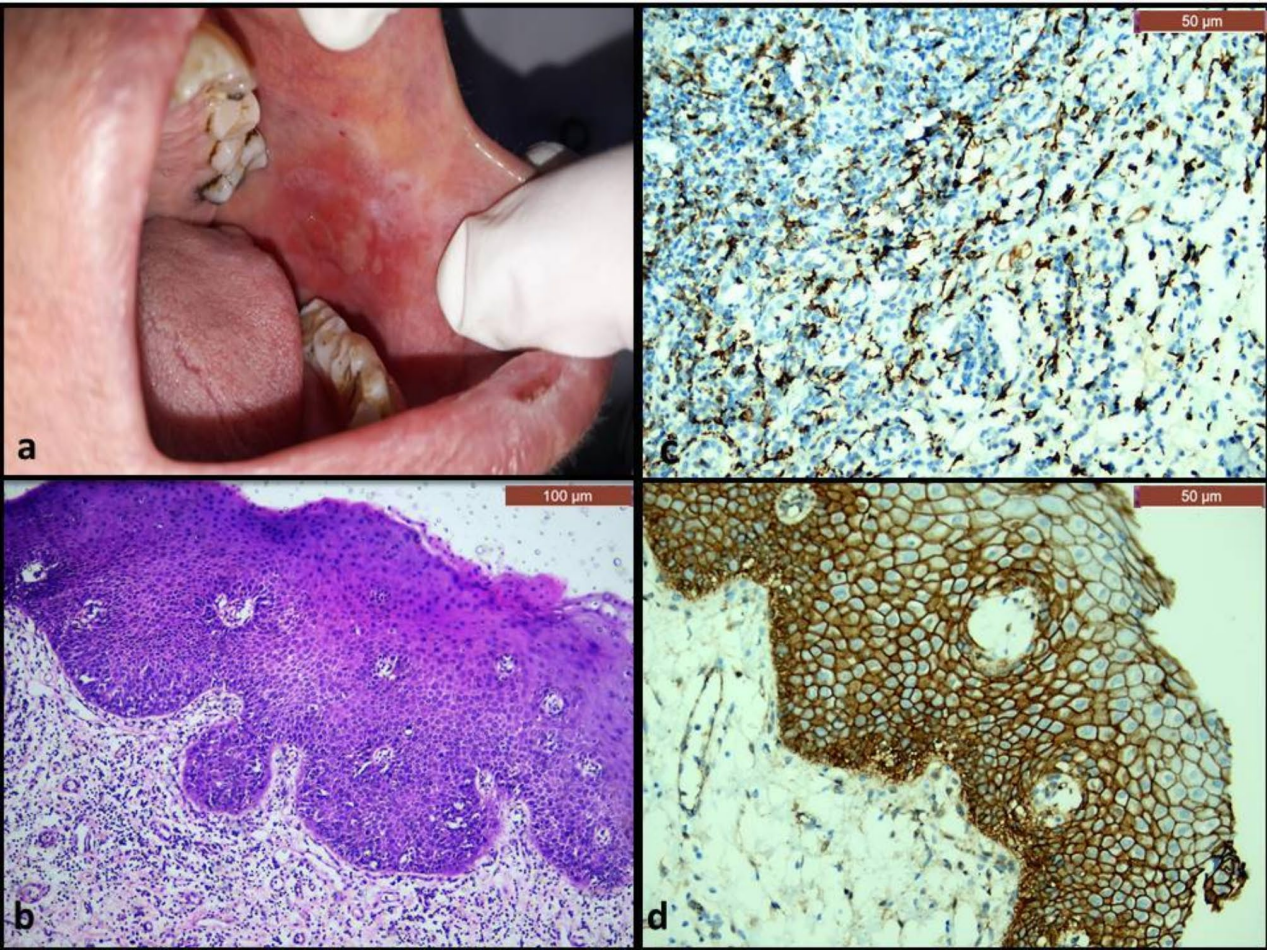
A case of dysplastic OLL: (**a**) clinical image showing ulcerative lesion on the buccal mucosa with tiny white areas. (**b**) Microscopic image showing hyperplastic stratified squamous epithelium which showed marked epithelial dysplasia with tear drop rete ridges (circle) (H&E,x100). (**c**) Immunohistochemical expression of CD163 showing cytoplasmic and nuclear expression in numerous cells in the connective tissue (x200). (**d**) Immunohistochemical expression of β-catenin showing strong membranous expression in epithelial cells with cytoplasmic expression in some cells in the basal two thirds of the epithelium, and nuclear expression in few basal cells (x200)

Histopathologically, all cases of OLP showed hyperplastic epithelium with thin rete ridges and Civatte bodies in the basal and parabasal cells (Fig. 2b). The epithelium was ulcerated in some areas, and showed dysplastic features in some cases (Fig. 4b). The underlying connective tissue showed sub-epithelial lymphocytic band that didn’t exceed the lamina propria (Figs. 2 and 4). On the other hand, all cases of OLL showed mixed inflammatory infiltrate that extended deeply in the submucosa, along with perivascular inflammation (Figs. 3 and 5). The dysplastic cases of OLP&OLL showed obvious architectural signs of epithelial dysplasia as basal cell clustering, expanded proliferative compartment of dysplastic epithelium, loss of cellular adhesions, bulbous rete ridges and generalized premature keratinization in single cells. In addition, many cytological dysplastic signs such as hyperchromatism, nuclear pleomorphism, karyomegaly, increase normal, abnormal mitotic figures and apoptotic mitosis were also detected in the histological sections of the dysplastic groups.

β-catenin expression in non-dysplastic OLP was membranous only (Fig. 2d), while, its expression in non-dysplastic OLL, dysplastic OLP and dysplastic OLL was membranous in the whole thickness of the epithelium. The cytoplasmic immune expression of β-catenin was confined in some basal and parabasal cells (Figs. 3, 4 and 5). Nuclear expression of β-catenin was detected in some of the dysplastic cases only (Figs. 4 and 5). Detailed distribution of β-catenin expression was summarized in Table 1. As for CD163 expression, it was nuclear and cytoplasmic in all the cases of the study groups.

**Table 1:** Showing a summary of β-catenin expression in the studied groups

Study groups	Area percent of β-catenin	Cytoplasmic expression		Nuclear expression	
		Percentage of cases	Mean count	Percentage of cases	Mean count
Non-dysplastic OLP	6.169%	0%	0	0%	0
Non-dysplastic OLL	22.289%	100%	38.2	0%	0
Dysplastic OLP	20.67%	60%	90.86	40%	8
DysplasticOLL	27.76%	100%	106.55	50%	6

**Fig. 6:**
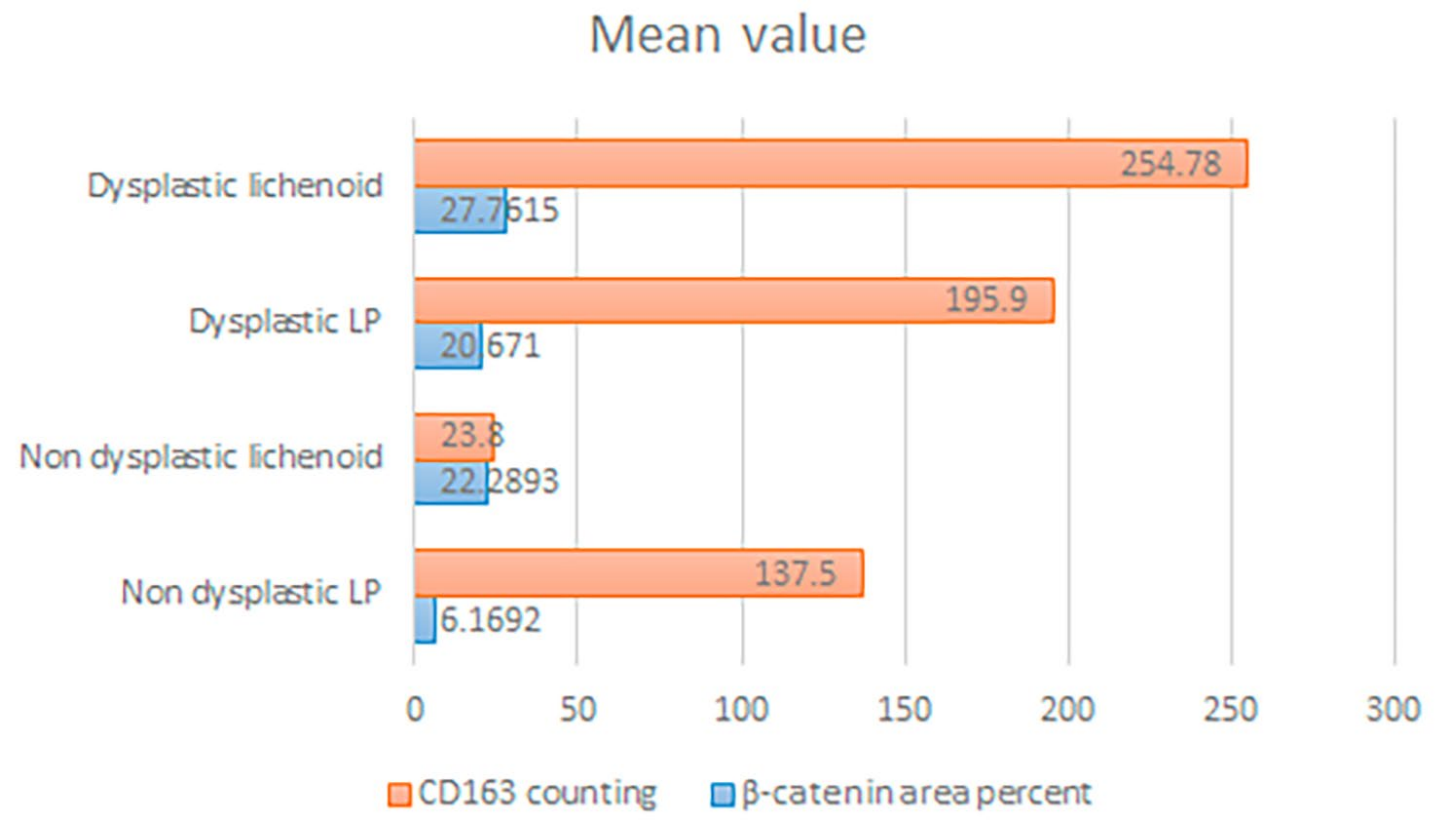
Clustered bar chart showing the mean area percent of β-catenin and CD163 count in the studied groups

After checking that the statistical data were normally distributed, we used a parametric statistical test (ANOVA test) for the statistical comparison between our study groups. The greatest mean of area percent of β-catenin expression in epithelium was recorded in dysplastic OLL group, non-dysplastic OLL and then dysplastic OLP, whereas the lowest value was recorded in non-dysplastic OLP group. One-way analysis of variance (ANOVA) test revealed that the difference between all groups was statistically significant (*P* < 0.0001). Tukey’s post hoc revealed significant differences among all groups except between dysplastic OLP and non-dysplastic OLL (Table 2; Fig. 6).

However, the greatest mean of CD163 count in stroma was recorded in the dysplastic OLL group, followed by dysplastic OLP and then non-dysplastic OLP, whereas the lowest value was recorded in the non-dysplastic OLL group. One-way analysis of variance (ANOVA) test revealed that the difference between all groups was statistically significant (*P* < 0.0001). Tukey’s post hoc revealed significant differences among all groups (Table 2; Fig. 6).

**Fig. 7:**
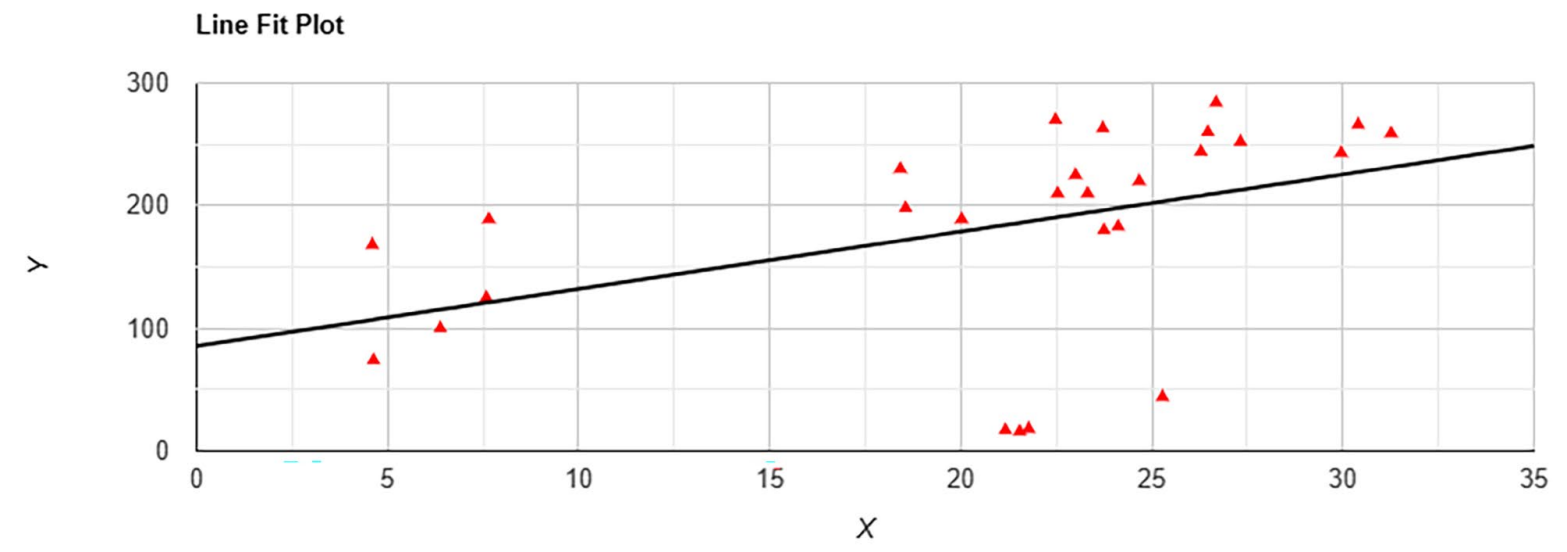
Line fit plot of Pearson correlation coefficient between area percent of β-catenin and CD163 count

Correlation between the area percent of β-catenin in epithelium and CD163 count in stroma was significant with moderate positive correlation; with correlation coefficient of *R* = 0.4 (*P* = 0.03) (Fig. 7).

**Table 2:** Showing a comparison between the mean area percent of β-catenin and CD163 count in the studied groups

Groups	area percent of β-catenin in the epithelium	Counting macrophages expressing (CD163)
Non dysplastic OLP	6.17 ± 1.5 ^d^	137.5 ± 45^c^
Non dysplastic OLL	22.29 ± 1.5 ^b, c^	23.8 ± 10.5^d^
Dysplastic OLP	20.67 ± 4.18 ^b, c^	195.9 ± 26.9^b^
Dysplastic OLL	27.76 ± 2.55 ^a^	254.78 ± 17.4^a^
P value	< 0.00001*	< 0.00001*

*Significant at *p* < 0.05

Tukey’s post hoc test: means sharing the same superscript letter are not significantly different.

## Discussion

OLP & OLL differ in etiopathogenesis and biological behavior; however, they mimic each other regarding the clinicopathological presentations. OLP is a chronic autoimmune mucocutaneous disease, which differs from OLL; as the second is usually caused by dental materials, an array of drugs, and graft versus, host diseases or unclassified causative agents as documented in the literature. Some histopathological differences were reported; such as higher number of apoptotic keratinocytes in OLL than in OLP, as well as more diffused and deeper sub-epithelial inflammation composed of mixture of plasma and eosinophils are usually reported in OLL cases [2]. In addition the significant increase in the maximum basement membrane thickness along with a focal increase in thickness in basement membrane can be considered as a diagnostic feature of OLP, whereas an increase in the intensity of degranulated mast cells deeper in the fibrous stromal tissues is more indicative of oral lichenoid reaction. Depending on the microscopic differences only is not enough to differentiate between OLP & OLL. Thus correlation between case history, clinical presentation and histopathology is mandatory to reach the final diagnosis [12].

Recently, OLP & OLL were reported as oral potentially malignant disorders (OPMDs) that can transform into OSCC [3]. Earlier some researchers have suggested that only OLLs, and not OLP, are OPMDs and should be classified as lichenoid dysplasia [1]. However, the latest edition of WHO stated that both conditions are considered as OPMDs [3]. Also we classified included OLP & OLL cases as dysplastic and non-dysplastic subcategories depending on the updated criteria of epithelial dysplasia in 5th edition of WHO [3]. The carcinogenesis of these lesions was not clearly explained, but it has been proposed that the production of chronic inflammatory mediators during development of these conditions can lead to accumulated genetic mutations and appearance of dysplastic epithelial changes, which may end up into frank malignant transformation [13].

Studies showed some micro-environmental factors, such as inflammatory cytokines cause impairment of the WNT pathway, which promotes OPMD transformation into malignancy. Upon Wnt cascade stimulation, β-catenin contributes as a target of it and finally controls cell replication and tumorgenesis [1, 14]. Activation of Wnt signaling will prevent β-catenin from ubiquitin-mediated decomposition phosphorylation, resulting in its aggregation in the cytoplasm & nucleus of the cell; this step will promote activation of oncogenes that stimulate cellular replication. Earlier studies suggested that β-catenin constitutes a hallmark of aggressive biological behavior of OSCC [15]. β-catenin is a transcriptional activator protein that belongs to the cadherin complex, which regulates the expression of several targets involved in cell development. Despite the alteration in Wnt/β-catenin pathway was reported to be involved in melanoma, gastric and cervical cancer, its relevance in OLP & OLL is not yet investigated according to our knowledge [16, 17].

It has been revealed that chronic inflammation of OLP causes mucosal damage, which eventually leads to the destruction of E-cad/ β-catenin combination and loss of the intercellular adhesions. It was previously documented in many studies that the membranous reduction of β-catenin may lead to its transfer and accumulation in the cytoplasm and the nucleus [14].

Earlier studies documented the reduction of membranous expression of β-catenin associated with the increase of its cytoplasmic and nuclear expression in dysplastic epithelial cells in OPMD & OSCC cases [18]. Our findings revealed that β-catenin immunoexpression, was found to be higher in non-dysplastic OLL cases than in OLP cases. Elevation of the cytoplasmic and nuclear expression of β-catenin increases the chance of malignant transformation of OLL than in OLP cases. On the same context, a previous study reported an elevation of nuclear/cytoplasmic expression of β-catenin to in cases of OSCC and dysplastic oral leukoplakia [19].

This could be explained as; the binding of E-cadherin to β-catenin at the epithelial cell membrane inhibits β-catenin accumulation in the cytoplasm, and also hinder its nuclear migration [20]. Consequently, it allows the formation of functional transcription factor with TCF/LEF-1 and subsequently, it prevents the activation of certain oncogenes. Therefore, its localization in the cytoplasm and/or the nucleus leads to the activation of certain oncogenes, and allows the progression of malignant transformation [4, 21].

On the other hand, Shigeoka et al., 2020 reported the significance of TAM expressing CD163 in tongue leukoplakia. As documented in cases of hepatocellular carcinoma, the tumor cells-derived Wnt ligands stimulate M2-like polarization via β-catenin signaling, which results in tumorigenesis. This revealed the potent role of TAM-specific β-catenin signaling in cancer progression. Also, they suggested a bidirectional role of β-catenin signaling in the crosstalk between tumor cells and TAMs [22–24]. Furthermore, transcriptional landscape enabled by β-catenin in cancer cells promotes vital cytokines genes activation & modulates the recruitment of M2 macrophages in the local tumor microenviroment [14].

Regarding our findings about the density of M2 macrophages (expressing CD163), the higher density was recorded to be higher in dysplastic cases than non-dysplastic cases of OLL and OLP cases. These findings were in accordance with a recent study that investigated CD163 expression in dysplastic OLL cases and reported a high density of M2 macrophages in such cases. They proposed that M2 could be a potent indicator for malignant transformation of OLL cases [4].

Another important study showed a significant elevation in density of CD163 + M2 macrophages in OPMD cases with moderate epithelial dysplasia compared to cases without epithelial dysplasia. They reported that M2 macrophages (expressing CD163+) were distributed in the sub-epithelial area. They explained this finding as M2 macrophages may be secreting matrix metalloproteinases, which promotes the destruction of basement membrane. A loss of basement membrane components has been correlated with the aggressiveness of some OPMD cases [24].

Thus the novelty of our presented study is to investigate the relation between CD163 + expression in M2 macrophages and also the epithelial expression of β-catenin in all OLP & OLL cases by Pearson Correlation Test, which proved a significant moderate positive correlation. Such a finding is not previously investigated in any other published studies until now. This was in accordance with earlier studies that suggested a correlation between M2 and stimulation of β-catenin nuclear/cytoplasmic expression in OPMD cases & some malignant cases [24, 25]. This correlation could be explained as Chen et al., 2022 proved that M2-macrophages can promote epithelial mesenchymal transition and cancer stem cells properties in β-catenin dependent manner, thus leading to cancer development [23]. This crucial finding will help the clinicians in the future in early cancer detection and prevention in cases of OLP& OLL. Depending on our reported results, we suggested using of both immune markers for prognostic prediction of dysplastic changes or malignant transformation in cases of OPMD. Another important implication is that both immune markers may be used as target therapy in such critical conditions (OPMD). Close monitoring of the suspected OLP & OLL cases by such immune markers will provide better guidance in follow up periods and treatment of such OPMD cases. Finally, we must clearly mention that the small number of sample size is considered a limitation in our study. This is attributed to the conservative treatment that is usually preferred for OLL& OLP cases rather than taking surgical biopsy for microscopic examination. Also we excluded any case with deficient medical history, which finally reduced the number of included cases in our study groups.

## Conclusion

In summary, our current study highlighted the contributing role of β-catenin signaling and CD163 + of M2 macrophages in the progression of epithelial dysplasia in cases of OPMDs. As well as, there is positive correlation between CD163 + M2 macrophages density & also the cytoplasmic and/or nuclear β-catenin expression. Our findings supported new perceptions for the malignant transformation mechanism of OLL & OLP. This may enhance the development of macrophage-based strategies for treatment of such cases. At the end we recommend studying the role of other cells in the tumor microenvironment of such important cases as often CD8-positive T lymphocytes or plasma cells in further studies. Also we recommend studying the degree of epithelial dysplasia in OPMD cases, by performing PCR technique for the β-catenin genetic expression on freshly taken oral biopsies and correlate the results with the β-catenin immunohistochemical analysis.

## Data Availability

No datasets were generated or analysed during the current study.

## Abbreviations

OLP: Oral lichen planus
WHO: World health organization
OSCC: Oral squamous cell carcinoma
OLL: Oral lichenoid lesion
OPMD: Oral potentially malignant disorder
TAM: Tumor associated macrophages
